# Blood Pressure Control and Mortality Among US Veterans

**DOI:** 10.1161/HYPERTENSIONAHA.125.25787

**Published:** 2026-04-07

**Authors:** Masaaki Yamada, Benjamin R. Griffin, Qianyi Shi, Meenakshi Sambharia, Melissa L. Swee, Mary K. Good, Korey Kennelty, Elissa Faro, Heather S. Reisinger, Saket Girotra, Brian C. Lund, Mary S. Vaughan Sarrazin, Diana I. Jalal

**Affiliations:** 1 Center for Access & Delivery Research and Evaluation, Iowa City VAMC, IA (M.Y., B.R.G., Q.S., M.L.S., M.K.G., H.S.R., B.C.L., M.S.V.S., D.I.J.).; 2 The University of Iowa Roy J. and Lucille A Carver, College of Medicine, IA (M.Y., B.R.G., Q.S., M.L.S., K.K., E.F., H.S.R., M.S.V.S., D.I.J.).; 3 Southwest Kidney Institute, Tempe, AZ (M.S.).; 4 The University of Iowa College of Pharmacy, Iowa City, IA (K.K.).; 5 The University of Texas Southwestern Medical Center, Dallas (S.G.).

**Keywords:** aged, blood pressure, hypertension, veterans

## Abstract

**BACKGROUND:**

Intensive blood pressure (BP) control reduces mortality and cardiovascular disease in clinical trials. However, real-world BP measurements often differ from standardized protocols. We evaluated the impact of real-world systolic BP on mortality among US Veterans.

**METHODS:**

We conducted a retrospective cohort study of Veterans with hypertension, defined by diagnostic codes, antihypertensive prescriptions, or ≥2 office BP readings ≥130/90 mm Hg in 2016 to 2017, with follow-up through March 2021. Systolic BP was treated as a time-dependent covariate and categorized into 7 groups: <110, 110−119, 120−129, 130−139, 140−149, 150−159, and ≥160 mm Hg. Discrete-time survival models assessed associations with all-cause mortality, adjusting for demographics, body mass index, and comorbidities. Stratified analyses were conducted based on cardiovascular disease and chronic kidney disease status.

**RESULTS:**

Among >2.3 million Veterans (mean age, 66 years; 36% with diabetes; 22% with cardiovascular disease; and 19% with chronic kidney disease), the lowest mortality risk was observed in those with systolic BP of 130 to 139 mm Hg. In this cohort, adjusted hazard ratios for all-cause mortality per year in each systolic BP category were 1.29 for BP <110; 1.03 for BP 110 to 119; 0.88 for BP 120 to 129; 0.83 for BP 130 to 139; 0.86 for BP 140 to 149; and 0.89 for BP 150 to 159 mm Hg, compared with a year with BP ≥160 mm Hg. These associations remained consistent across cardiovascular disease and chronic kidney disease subgroups.

**CONCLUSIONS:**

Veterans with routine systolic BP of 130 to 139 mm Hg had the lowest mortality. These findings suggest that a higher BP target may be appropriate in clinical practice, especially for older adults with comorbidities.

**Novelty and Relevance**

**What Is New?**

This study uses real-world data from over 2.3 million US Veterans to examine the association between routinely measured systolic blood pressure (BP) and all-cause mortality. Unlike randomized controlled trials that use standardized BP protocols under controlled conditions, our analysis reflects routine office BP measurements, which are subject to variability in technique, timing, and clinical context. This approach provides insights into BP targets for a high-risk, older population with multiple comorbidities.

**What Is Relevant?**

Observational studies capture the complexity of real-world practice in the management of hypertension, including measurement variability and patient heterogeneity. Our findings suggest that systolic BP 130 to 139 mm Hg in routine care is associated with the lowest mortality risk.

**Clinical/Pathophysiological Implications?**

These results emphasize the need for flexibility in interpreting clinical guidelines. Additionally, to implement intensive BP control as recommended by the guidelines and supported by large clinical trials, efforts are needed to standardize BP measurement.

The potential benefits of intensive blood pressure (BP) control (systolic BP <120−130 mm Hg) were suggested in a post hoc analysis of the 2010 ACCORD BP trial (Action to Control Cardiovascular Risk in Diabetes Blood Pressure).^1^ Later, the beneficial cardiovascular effects were confirmed by the 2015 SPRINT (Systolic Blood Pressure Intervention Trial) in individuals without diabetes.^2^ Subsequent trials and meta-analyses have further established the case for the intensive systolic BP control,^3–5^ leading several medical societies to revise the definition of hypertension and BP control treatment goals.^6–8^

To achieve adequate BP control under the recently revised, more stringent treatment goals, medication intensification is essential. While this can be safely done in clinical trials, it is challenging in routine clinical practice, where resources are limited and therapeutic inertia is prevalent.^9–12^ A major factor that contributes to therapeutic inertia among clinicians is uncertainty about the diagnosis of uncontrolled hypertension and low confidence in clinic BP measurements due to both visit-to-visit variability in BP values^13^ and potential inaccuracies in outpatient clinic BP measurement.^14,15^ These are valid concerns since the trials that have established intensive BP management did so based on standardized BP measurements in the research setting.

Patient factors also contribute to suboptimal BP control. Veterans, for example, represent an older patient population with a high prevalence of multiple comorbidities. The complexities of polypharmacy and concerns about potential side effects are particularly significant in this group.^16,17^ These differences in patient factors can contribute to an implementation gap in evidence-based practices. The analysis of real-world data is crucial to apply clinical research findings to the broader patient population seen in routine clinical practice.^18^ While some observational data have evaluated this among Veterans with chronic kidney disease (CKD), no data exist for the general population of Veterans. Considering that standardized measurement of outpatient clinic BP, as performed in clinical trials, is often lacking in routine clinical practice, it is critical to assess the achieved BP values of these Veterans with a higher burden of comorbidities.^19^ Here, we sought to investigate the impact of real-world systolic BP values measured in clinic offices on clinical outcomes among Veterans.

## Methods

### Data Availability

The data used in this study are derived from the US Department of Veterans Affairs (VA) electronic health records and are not publicly available due to federal privacy regulations and VA data use policies. They may be made available from the corresponding author upon reasonable request and with permission from the VA. However, access to these data requires appropriate VA credentials, institutional review board approval, and a Data Use Agreement, as outlined in Veterans Health Administration (VHA) Directive 1080.01(1).

### Data Source and Study Population

This study utilized data from the VHA national patient care database, which includes comprehensive outpatient and inpatient medical records for all health care encounters within the VHA system. The institutional review board and Research and Development Committee at the Iowa City VA Health Care System (institutional review board identification number 202008361) have approved this study and waived informed consent.

We conducted a retrospective cohort study of Veterans with prevalent hypertension identified between January 1, 2016 and December 31, 2017. For inclusion in the study, hypertension was defined as meeting ≥1 of the following criteria: ≥2 episodes of elevated office BP of ≥130/90 mm Hg, a relevant hypertension diagnosis based on the *International Classification of Disease-Tenth Revision*, Clinical Modification, or receipt of any first-line antihypertensive medication therapy in accordance with the VHA and American College of Cardiology/American Heart Association guidelines.^6,7,20–22^ A 1-year baseline period was established for each patient, beginning from the first observed hypertension diagnosis or antihypertensive medication refill during the 2016 to 2017 identification window. During this period, we assessed baseline comorbidities and BP categories. Antihypertensive medication exposure at baseline was determined using VA pharmacy dispensation records. Medications were categorized by mechanism of action, including angiotensin-converting enzyme inhibitors or angiotensin receptor blockers, β-blockers, dihydropyridine calcium channel blockers, thiazide or thiazide-type diuretics, potassium-sparing diuretics, and aldosterone antagonists. A detailed list of included medications is provided in Table S1. Veterans were excluded if they had a history of dementia, metastatic cancer, severe liver disease, end-stage kidney disease, or were on palliative status.^22^ In addition, Veterans using midodrine or those with mean baseline systolic BP <100 mm Hg were excluded to minimize reverse causation that could bias the results.^23^ The cohort follow-up period began 1 year after the index date, following the baseline ascertainment period. Veterans who died during the baseline period were excluded from the analysis (Figure). All eligible Veterans were followed from the start of follow-up through March 31, 2021.

**Figure.**
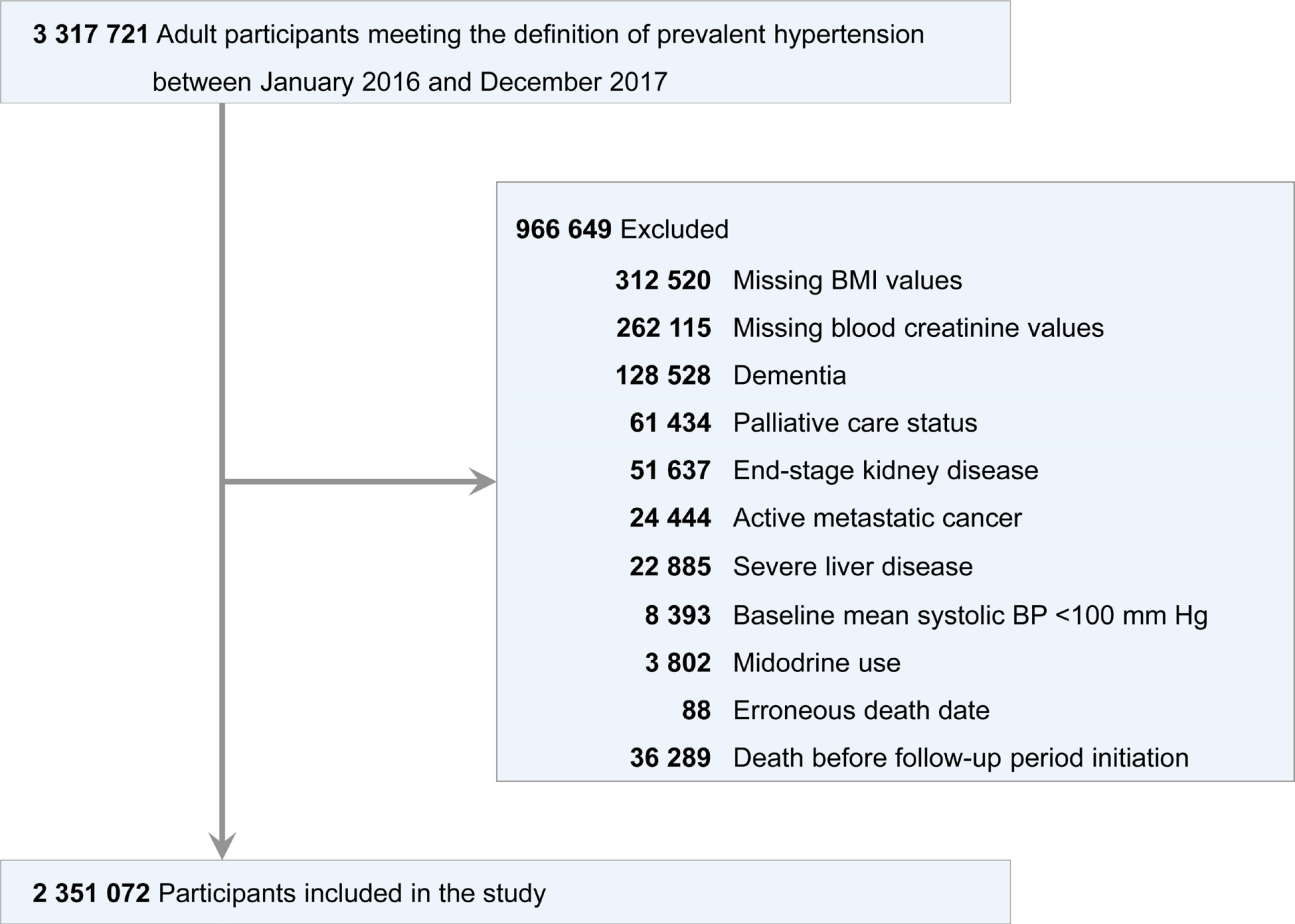
**Cohort study patient flowchart.** We identified 3 317 721 adults with prevalent hypertension between January 2016 and December 2017. After exclusions (n=966 649) for missing data and baseline conditions (eg, missing body mass index [BMI], missing creatinine, dementia, palliative care, end-stage kidney disease, active metastatic cancer, severe liver disease, baseline systolic blood pressure (BP) <100 mm Hg, midodrine use, erroneous death date, or death before follow-up initiation), 2 351 072 participants were included in the final study cohort.

### BP Measurements

BP values were included if they were recorded during outpatient visits.^17^ We excluded nonphysiological values (ie, systolic BP <40 mm Hg or >300 mm Hg; diastolic BP <10 mm Hg; and systolic BP less than diastolic BP).^17,24,25^ If multiple readings were recorded for a single date, we used the lowest systolic and lowest diastolic BP values as representative in the analysis.

### BP Categories

Systolic BP measures were treated as time-dependent and categorized as <110, 110 to 119, 120 to 129, 130 to 139, 140 to 149, 150 to 159, and ≥160 mm Hg, with time in each category accumulated in 30-day increments. We focused on systolic BP as the predictor, consistent with the current VA/Department of Defense Clinical Practice Guideline that recommends intensive systolic but not diastolic BP lowering (target diastolic BP remains <90 mm Hg).^7^ In addition, based on our previous work, we have found that among Veterans with diastolic BP ≥80 mm Hg, only 5% had a diastolic BP ≥ 90 mm Hg.^17^

### Primary Outcome

Our outcome was all-cause mortality through March 31, 2021. The mortality data were derived from the VA Corporate Data Warehouse, which includes mortality information from the Veterans Benefits Administration and the Social Security Administration.

### Covariables

Baseline demographic data and clinical characteristics included in the analysis were obtained at the time of first BP measurements. The following baseline variables were included in the analysis: age (years), sex (male, female), race (White, Black, Other), and body mass index, which was calculated from height and weight measurements (kg/m^2^). The social vulnerability index was derived from 2018 Census data and expressed in quantiles (Q1–Q4), with Q1 representing the least vulnerable and Q4 the most vulnerable. This allowed for the assessment of health disparities and social determinants of health.^26,27^ Baseline comorbidities were defined using the Elixhauser comorbidity index.^20,22^ Cardiovascular-related diagnoses, namely, coronary artery disease (CAD), prior revascularization, and congestive heart failure, were grouped as CVD.^28^ To accurately assess kidney dysfunction, the estimated glomerular filtration rate was calculated using the 2021 creatinine-based equation.^29^

### Statistical Analysis

We used discrete-time survival models to examine the association of systolic BP control with all-cause mortality. This method performs similarly to Cox proportional hazards regression but involves less complex estimation in the presence of time-dependent covariates.^30,31^ The model estimates the relative chance of death in the next follow-up interval among patients still at risk. Using this approach, the follow-up period for each patient was segmented into monthly intervals (defined as 30.5 days) through the date of the outcome or censoring event (whichever came first), allowing for a maximum of 48 possible follow-up intervals per patient.^31^ Systolic BP was treated as time-dependent by allowing time spent within each BP category to accumulate over sequential months. The model estimated the relative hazards of all-cause mortality per cumulative month spent in each BP category, relative to cumulative months with BP ≥160 mm Hg. Results were expressed as the hazard ratio per 12 months in each BP category, compared with 12 months with BP ≥160 mm Hg. The model adjusted for baseline demographics, body mass index, estimated glomerular filtration rate, and comorbid conditions defined by diagnostic codes. For variable selection, stepwise approaches with forward selection and backward elimination were used, along with clinical judgment and literature, to achieve a parsimonious model. Models used robust standard errors with an exchangeable working correlation matrix to account for multiple observations per patient over time. We also fitted a traditional Cox proportional hazards model that treated systolic BP as time-dependent. This analysis used a random 20% subsample, and we repeated the discrete-time survival model in the same subsample; details are in Table S2. A 2-sided *P* value of 0.05 was considered statistically significant, and 95% CIs were presented for all point estimates. Additional sensitivity analyses were performed based on the presence or absence of a history of CVD and the stage of CKD, as these factors are known to influence BP control and are associated with clinical outcomes. Analyses were conducted with SAS software version 9.4 (SAS Institute, Cary, NC).

## Results

### Baseline Characteristics

During 2016 to 2017, a total of 2 351 072 hypertensive Veterans were eligible and included in our study (Figure). The mean age (SD) of this cohort was 65.7 (12.3) years, and it was predominantly male (94.6%) and White (71.3%; Table 1). A substantial portion of hypertensive Veterans, comprising 66.7% (n=1 567 274), had mean systolic BP levels ≥130 mm Hg at baseline, which is considered uncontrolled according to recently revised clinical practice guidelines. In this cohort, 37.0% of Veterans (n=871 045) had diabetes, 21.7% (n=509 693) had a prior history of CVD, 7.4% (n=174 181) had atrial fibrillation, 6.7% (n=158 107) had peripheral artery disease, and about 18.9% (n=444 629) had CKD defined by estimated glomerular filtration rate <60 mL/min per 1.73 m^2^. In addition to the cardiovascular-related conditions, there was a significant number of Veterans with chronic lung disease (n=372 047; 15.8%).

**Table 1.**
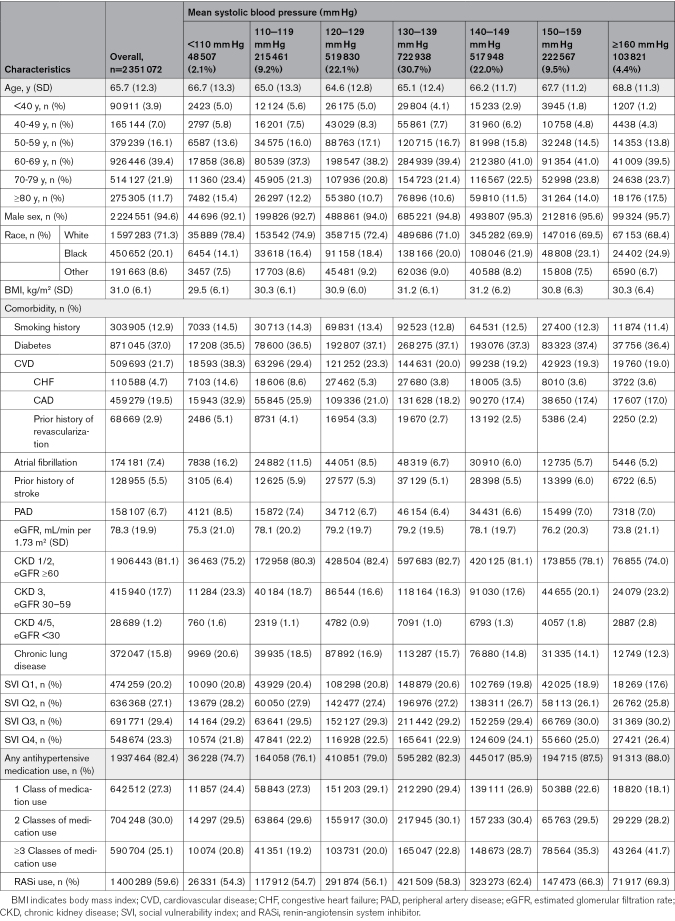
Baseline Characteristics

We then evaluated the demographics of our cohort based on baseline systolic BP categories defined as: <110, 110 to 119, 120 to 129, 130 to 139, 140 to 149, 150 to 159, and ≥160 mm Hg. The <110 mm Hg group had a higher representation of White individuals (78.4%) and lower representation of Black individuals (14.1%) compared with the ≥160 mm Hg group (68.4% White, 24.9% Black; Table 1). The prevalence of diabetes was similar across systolic BP categories, ranging from 35.5% to 37.5%. In contrast, the prevalence of CVD was highest in the <110 mm Hg group (38.5%) and lowest in the ≥160 mm Hg group (19.1%).

Overall, 82.4% of the Veterans (n=1 941 251) were prescribed at least 1 class of antihypertensive medication at baseline (Table 1). Single-class medication use was relatively consistent across the systolic BP categories below 140 mm Hg (24.6%–29.9%) but declined to 18.5% with systolic BP ≥160 mm Hg. Two-class medication use remains stable across all systolic BP categories (30.0%–30.9%). The use of >3 medication classes increased with higher systolic BP, from 18.5% to 20.2% in the <120 mm Hg group to 40.5% in the ≥160 mm Hg group. Renin-angiotensin system inhibitor use was highest in the ≥160 mm Hg group (69.3%) followed by 66.3% in the 150 to 159 mm Hg group, 62.4% in the 140 to 149 mm Hg group, and lowest in the <110 mm Hg group (54.3%; *P*<0.001).

### Risk of Death According to BP Categories

The overall mortality was 5.43 deaths per 100 patient-years (95% CI, 5.41–5.44). Patients in the baseline systolic BP <110 mm Hg group had the highest mortality, at 8.92 deaths per 100 patient-years (95% CI, 8.78−9.06). For Veterans with a mean baseline systolic BP of 110 to 119, 120 to 129, 130 to 139, 140 to 149, 150 to 159, and ≥160 mm Hg, the mortality rates (95% CI) were 6.29 (6.23−6.34), 5.12 (5.09−5.15), 4.82 (4.80−4.85), 5.19 (5.16−5.22), 6.17 (6.12−6.22), and 7.60 (7.51−7.68) deaths per 100 patient-years, respectively. In discrete-time survival models with time-dependent systolic BP, the adjusted hazard ratios (95% CI) for all-cause mortality were 1.29 (1.28−1.31) per 12 months with BP <110 mm Hg, 1.03 (1.02−1.04) per 12 months with BP 110 to 119 mm Hg, 0.88 (0.87−0.89) per 12 months with BP 120 to 129 mm Hg, 0.83 (0.82−0.84) per 12 months with BP 130 to 139 mm Hg, 0.86 (0.85−0.87) per 12 months with BP 140−149 mm Hg, and 0.89 (0.88−0.90) per 12 months with BP 150−159 mm Hg, compared with 12 months with BP ≥160 mm Hg (Table 2).

**Table 2.**
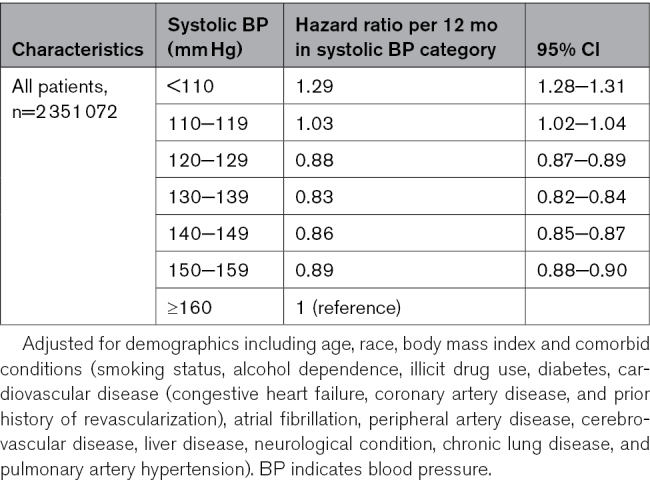
Hazard Ratios (95% CIs) of All-Cause Mortality Associated With Time-Varying BP Categories

### Sensitivity Analysis

Considering the significant differences in comorbidity, we explored the potential interaction between BP category and age ≥65 years, history of diabetes, and CVD, which increase cardiovascular risk. These interaction terms were not significant for any of the variables. We also tested for time-by-systolic BP category interactions to determine whether relative hazards were proportional over time; results did not indicate nonproportionality of relative hazards. Table 3 presents the all-cause mortality risk associated with time in different systolic BP categories for the subgroups with and without CVD. Notably, for Veterans with no history of CVD, the hazard ratio (95% CI) was highest at 1.29 (1.27−1.31) for systolic BP <110 mm Hg. The hazard ratio gradually declined to a minimum of 0.81 (0.81–0.82) in the 130−139 mm Hg range, and then increased again toward the reference systolic group (≥160 mm Hg). This J-shaped association was consistently observed across various subgroups, including Veterans with CVD, those with and without CKD history, and sex-specific subgroups (male-only and female-only Veterans), as shown in Tables 3 through 5. A time-varying Cox model estimated in a 20% random sample yielded hazard ratios closely aligned with the discrete-time analysis; details are shown in Table S2.

**Table 3.**
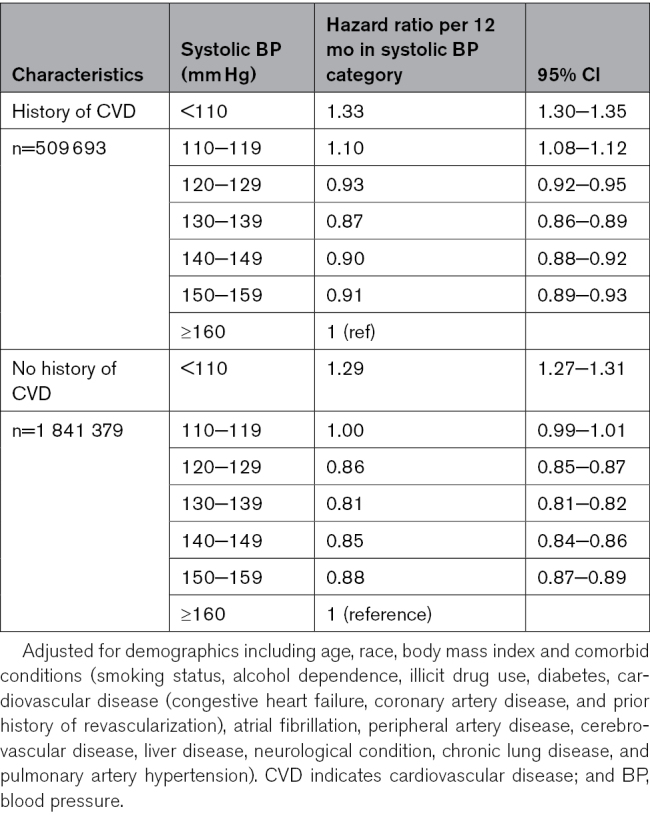
Hazard Ratios (95% CIs) of All-Cause Mortality Associated With Time-Varying BP Categories Stratified by CVD History

**Table 4.**
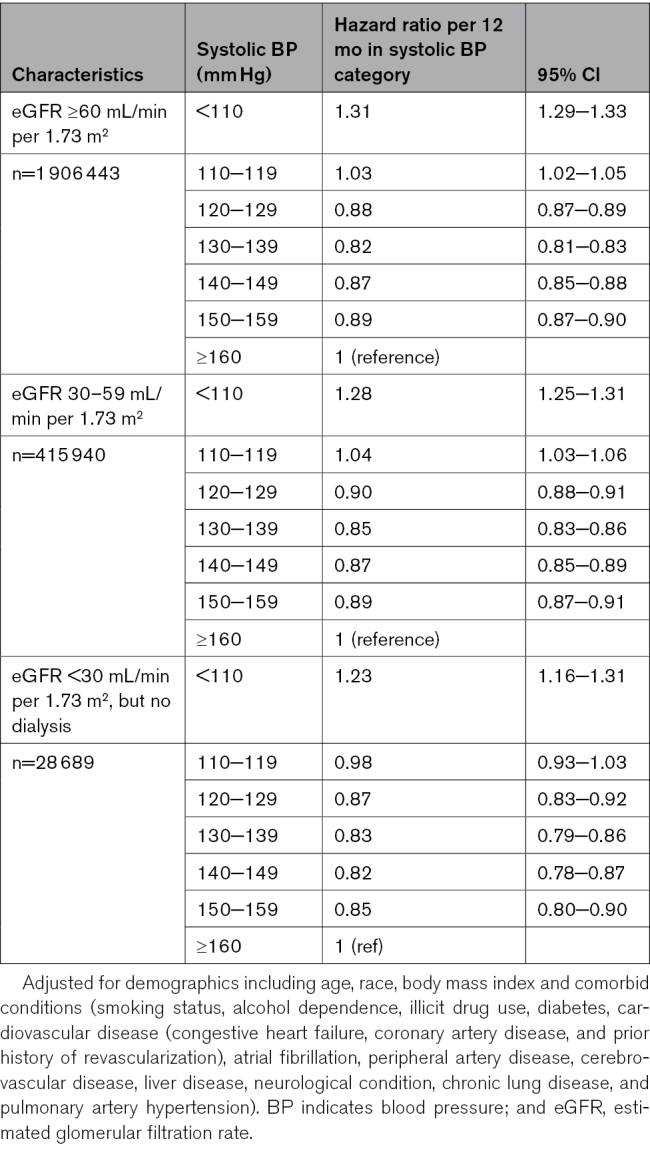
Hazard Ratios (95% CIs) of All-Cause Mortality Associated With Time-Varying BP Categories Stratified by Kidney Function

**Table 5.**
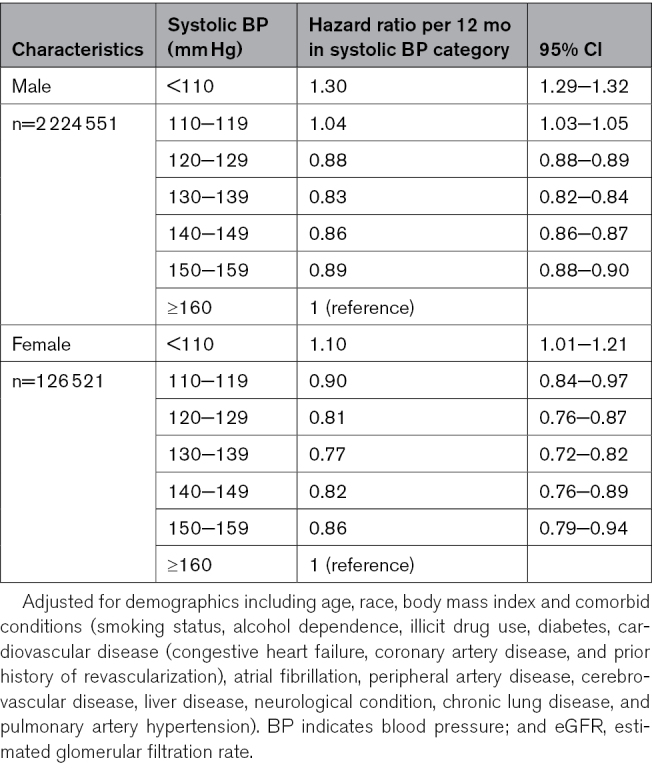
Hazard Ratios (95% CIs) of All-Cause Mortality Associated With Time-Varying BP Categories Stratified by Sex

## Discussion

Our analysis of data from over 2.3 million hypertensive Veterans suggests that maintaining systolic BP within the range of 130 to 139 mm Hg, based on routine office measurements, is associated with the lowest mortality risk, compared with systolic BP of ≥160 mm Hg. One year spent with systolic BP 120 to 129 mm Hg, 130 to 139 mm Hg, and 140 to 149 mm Hg was associated with 12%, 17%, and 14% lower risk of death, respectively, compared with 1 year with systolic BP ≥160 mm Hg. This pattern reflects a J-shaped relationship, where mortality risk increases at both high and very low BP values. Importantly, these findings were independent of preexisting CVD and CKD, and our findings are consistent with the results of previous observational studies.^1,2,32^ The observation that the lowest risk of all-cause mortality was noted with systolic BP 130 to 139 mm Hg suggests that more careful consideration is necessary when implementing intensive BP lowering based on routine office BP values in clinical practice.

Several factors may contribute to the differential findings between observational and interventional studies. First, clinical trial protocols create ideal conditions for participant evaluation that are difficult to replicate in routine practice. Most relevant here is standardized BP measurement. In regular clinical practice, BP measurement is not always standardized. Based on a post hoc analysis of the SPRINT data, routine clinic BP measurements averaged 4 to 7 mm Hg higher than automated unattended office BP measurements.^33^ Not only are routine clinic BP measurements higher than standardized measurements, but there is also considerable visit-to-visit variation in routine clinic BP, further complicating the interpretation of routine office BP values.^13^ Visit-to-visit BP variation occurs due to inconsistencies in the timing and context of office BP measurement, the use of different measurement devices, and the setting (eg, medication use and timing of visits) in which BP is measured. The use of standardized BP measurements, as performed in clinical trials, is ideal, but its widespread use is challenging to replicate in routine practice due to the significant time and resources required. Consequently, the limitations of routine clinic BP measurements make it difficult to apply clinical trial findings and implement guideline-recommended approaches effectively in everyday clinical practice.

In addition to differences in study design between observational and clinical trials, another plausible explanation for the lowest mortality risk being associated with 130 to 139 mm Hg (as opposed to 120−129 mm Hg) is that there are notable differences in patient characteristics between real-life patients and clinical trial participants.^34^ Our findings align with the results of previous observational studies. For example, Vidal-Petiot et al,^35^ in a large prospective longitudinal observational cohort study of 22 672 patients with stable CAD treated for hypertension (CLARIFY [Prospective observational longitudinal regIstry of patients with stable coronary artery disease] registry), reported that a systolic BP in the range of 120 to 140 mm Hg was associated with the lowest mortality risk. Similarly, a retrospective study by Navaneethan et al^36^ using data from the Cleveland Clinic CKD registry found that patients with CKD who maintained systolic BP between 130 and 139 mm Hg had the lowest mortality risk. In our cohort, the annual mortality rate was 5.5%, compared with 8.3% in the CLARIFY registry, and 7.6% in the Cleveland Clinic CKD registry. These rates are substantially higher than those reported in randomized controlled trials such as SPRINT (1−1.4%) and the ACCORD BP trial (1.2−1.3%). The elevated mortality rates observed in these observational studies likely reflect differences in patient characteristics and disease severity in real-world settings compared with clinical trials. Notably, 74.8% (n=1 761 681) of our cohort possessed at least 1 high cardiovascular risk factor. These findings underscore the importance of interpreting clinical trial data with caution and considering the broader context of real-world clinical practice.

Finally, intensive lowering of systolic BP also lowers diastolic BP, and low diastolic BP may impair coronary perfusion. This may contribute to an increased risk of cardiovascular events and death especially among individuals with CAD. For instance, evidence from the CLARIFY registry, which included over 22 000 patients with stable CAD, showed that both systolic BP <120 mm Hg and diastolic BP <70 mm Hg were associated with increased cardiovascular risk.^35^ Similarly, the MESA (Multi-Ethnic Study of Atherosclerosis), which followed over 6800 participants without known CVD, found that diastolic BP <60 mm Hg was linked to higher risks of coronary heart disease and all-cause mortality.^37^ This association was most pronounced among individuals with severe coronary artery calcification, suggesting that an excessively low diastolic BP may impair coronary perfusion even in the absence of symptomatic CAD. It should be noted that the evidence supporting increased rates of mortality with low diastolic BP is conflicted. For example, data by Beddhu et al^38^ from SPRINT indicate that the benefits of intensive lowering of systolic BP were evident even among those with diastolic BP <68 mm Hg. This remains an area of active research, underscoring the complexity of identifying optimal BP targets in diverse patient populations.

Our study has several limitations. First, despite the valuable insights gained from our study and other observational studies, they cannot establish causality or fully explore the underlying pathophysiologic mechanisms driving the observed differences. Furthermore, while we made efforts to adjust for potential confounders by leveraging the large-scale data set, some residual confounding may persist. In a sensitivity analysis, we repeated the analysis using a time-varying Cox model in a random 20% sample; estimates were essentially the same, and the J-shaped association persisted (Table S2), suggesting the findings are not model-dependent. We also acknowledge the possibility of reverse causation, where lower BP measurements may reflect end-of-life processes. We sought to minimize this bias by excluding inpatient BP values and individuals with average systolic BP <100 mm Hg at baseline and certain comorbid conditions (advanced liver disease, malignancy, and hospice status). However, it is possible we did not entirely eliminate reverse causation in our study design. Second, our study cohort predominantly consisted of elderly White individuals, with a small proportion of women, reflecting the demographics of the VHA data set. This demographic composition may limit the generalizability of our research findings to more diverse populations. Third, there might be potential misclassification of prevalent hypertension due to medications used for nonhypertension indications (eg, β-blockers for CAD or atrial fibrillation). However, we limited our analysis to the first-line therapeutic medications only, excluding β-blockers, to identify our prevalent hypertensive cohort. This effort minimizes the misclassification bias from the use of β-blockers for nonhypertensive indications. We also performed sensitivity analyses to ensure that our findings were replicated in these patients without prevalent CAD or CKD. We did not account for incident comorbidities that developed during follow-up, which may influence our results. Because such conditions often lie on the causal pathway linking BP to mortality (eg, uncontrolled BP may contribute to heart failure progression and subsequent mortality), adjusting for their development could introduce bias. Finally, we did not include antihypertensive medications in the adjusted models, as estimating precise effects of medication usage in a retrospective study is challenging. Furthermore, the prior study by Kovesdy et al^25^ demonstrated that the addition of antihypertensive medications did not significantly change effect estimates, indicating that antihypertensive medication usage was not a clinically significant explanatory variable.

## Conclusions

The findings of our study, which included over 2.3 million of hypertensive Veterans, carry significant implications for individuals with hypertension, health care clinicians, and society at large. First, lower office systolic BP was consistently associated with reduced mortality among Veterans with hypertension, regardless of their history of CVD or CKD. Second, an achieved systolic BP of 130 to 139 mm Hg, based on the office BP measurements, was associated with the lowest mortality risk among hypertensive Veterans. These data highlight the limited applicability of guideline-recommended BP targets derived from clinical trials to real-world patients and health care systems. Initiatives to standardize BP measurement in routine clinical practice are needed to implement intensive BP management. Concurrently, some flexibility in guideline interpretation is warranted for older adults with a high burden of comorbid conditions who are likely excluded from the trials.

## Perspectives

Our study complements evidence from randomized controlled trials by evaluating the association between BP categories as measured in real-world clinical practice. Here, we observed a J-shaped association between systolic BP and mortality, with the lowest mortality rates in the category of systolic BP 130 to 139 mm Hg. The current guidelines, based on large randomized controlled trials, support intensive BP control (systolic BP <130 mm Hg). Our findings may be explained by differences in study design. Randomized controlled trials use standardized BP measurement protocols and enroll highly selected populations, whereas routine care involves variable measurement techniques and patients with multiple comorbidities. These findings suggest that some flexibility may be warranted when implementing clinical guidelines in real-world settings.

## Article Information

### Acknowledgments

The authors like to express their sincere gratitude to the members of the Center for Access and Delivery Research & Evaluation (CADRE) for their invaluable support and collaboration throughout this study. In particular, we are deeply thankful to Dr Peter J. Kaboli, Dr Michael P. Jones, and Dr Hiroyuki Suzuki for their insightful guidance, encouragement, and contributions to the development and refinement of this work. This work was done at the Center for Access & Delivery Research and Evaluation at the Iowa City VA Health Care System.

### Disclosures

None.

## Supplementary Material
