# Supplementary material for: Blood Pressure Control and Mortality Among US Veterans
Source: Hypertension. 2026 Apr 7;83(6):e25787. doi: 10.1161/HYPERTENSIONAHA.125.25787 (PMC13189391; doi:10.1161/HYPERTENSIONAHA.125.25787)
Supplement: Supplementary file 1 [file hyp-83-e25787-s001.docx]

**SUPPLEMENTAL MATERIAL**

**Title: Blood Pressure Control and Mortality among U.S. Veterans**

**Author:** Masaaki Yamada, MD; Benjamin R. Griffin, MD; Qianyi Shi, PhD; Meenakshi Sambharia, MD; Melissa L. Swee, MD; Mary K. Good, PhD; Korey Kennelty, PharmD; Elissa Faro, PhD; Heather Reisinger, PhD; Saket Girotra, MD; Brian C. Lund, PharmD, MS; Mary S. Vaughan Sarrazin, PhD; Diana I. Jalal, MD

**Corresponding Author:** Masaaki Yamada, M.D.
Street Address: 200 Hawkins Dr., Division of Nephrology, E300C GH, Iowa City, IA 52242
Corresponding Author’s Email Address: masaaki-yamada@uiowa.edu

**1. Supplemental Tables**

**Supplementary Table S1.** Medications included in the analysis

**Supplementary Table S2.** Hazard ratios for all‑cause mortality per 12 months across systolic blood pressure categories: comparison of time‑varying Cox regression and discrete‑time survival analyses in a 20% random sample and the full cohort

**Supplementary Table S1.** Medications included in the analysis

| Medication Class | Medication Generic Name |
| --- | --- |
| Thiazide or thiazide-type diuretics | Chlorthalidone, Hydrochlorothiazide, Indapamide, Metolazone |
| Aldosterone antagonists | Eplerenone, Spironolactone |
| Potassium-sparing diuretics | Amiloride, Triamterene |
| Angiotensin-converting enzyme inhibitors | Benazepril, Captopril, Enalapril, Fosinopril, Lisinopril, Moexipril, Perindopril, Quinapril, Ramipril, Tarndolapril |
| Angiotensin 2 Receptor blockers | Azilsartan, Candesartan, Eprosartan, Irbesartan, Losartan, Olmesartan, Telmisartan, Valsartan |
| Calcium channel blockers-dihydropyridines | Amlodipine, Felodipine, Isradipine, Nicardipine, Nifedipine, Nisoldipine |
| Calcium channel blockers-  nondihydropyridines | Diltiazem, Verapamil |
| Beta blockers | Atenolol, Betaxolol, Bisoprolol, Metoprolol Tartrate, Metoprolol Succinate, Nebivolol, Nadolol, Propranolol, Acebutolol, Penbutolol, Pindolol, Carvedilol, Labetalol |

**Supplementary Table S2.** Hazard ratios for all‑cause mortality per 12 months across systolic blood pressure categories: comparison of time‑varying Cox regression and discrete‑time survival analyses in a 20% random sample and the full cohort

| Systolic BP Category* (mm Hg) | Traditional Cox regression** (20% random sample) | Discrete-time survival analysis (20% random sample) | Discrete-time survival analysis (Full cohort) |
| --- | --- | --- | --- |
|  | HR (95% CI) | HR (95% CI) | HR (95% CI) |
| <110 | 1.31 (1.28-1.34) | 1.29 (1.26-1.33) | 1.29 (1.28−1.31) |
| 110-119 | 1.01 (0.99-1.04) | 1.04 (1.01-1.06) | 1.03 (1.02−1.04) |
| 120-129 | 0.87 (0.85-0.88) | 0.87 (0.85-0.89) | 0.88 (0.87−0.89) |
| 130-139 | 0.83 (0.81-0.84) | 0.84 (0.82-0.86) | 0.83 (0.82−0.84) |
| 140-149 | 0.85 (0.83-0.87) | 0.87 (0.85-0.89) | 0.86 (0.85−0.87) |
| 150-159 | 0.87 (0.85-0.90) | 0.88 (0.85-0.90) | 0.89 (0.88−0.90) |
| ≥160 | Reference | Reference | Reference |
| *Per 12 months spent in each systolic BP category; **Traditional Cox regression model estimated time to death in days and treated systolic BP category as a time-dependent covariate; HR, hazard ratio; CI, confidence interval | | | |
